# Minimum distance quantile regression for spatial autoregressive panel data models with fixed effects

**DOI:** 10.1371/journal.pone.0261144

**Published:** 2021-12-14

**Authors:** Xiaowen Dai, Libin Jin

**Affiliations:** 1 School of Statistics and Mathematics, Shanghai Lixin University of Accounting and Finance, Shanghai, China; 2 Interdisciplinary Research Institute of Data Science, Shanghai Lixin University of Accounting and Finance, Shanghai, China; Yunnan University of Finance and Economics, CHINA

## Abstract

This paper considers the quantile regression model with individual fixed effects for spatial panel data. Efficient minimum distance quantile regression estimators based on instrumental variable (IV) method are proposed for parameter estimation. The proposed estimator is computational fast compared with the IV-FEQR estimator proposed by Dai et al. (2020). Asymptotic properties of the proposed estimators are also established. Simulations are conducted to study the performance of the proposed method. Finally, we illustrate our methodologies using a cigarettes demand data set.

## 1 Introduction

In the last few decades, spatial autoregressive (SAR) models have been studied and applied to many areas such as economics, demography, geography and other scientific areas. Panel data with spatial interaction is also of great interest, as it can control for both heterogeneity and spatial correlation and enable researchers to take into account the dynamics (see, [1–7]).

Recently, there has been a growing literature on estimating and testing of spatial panel data models. For instance, [7] proposed the maximum likelihood (ML) estimator for the spatial autoregressive (SAR) panel model with both spatial lag and spatial disturbances. Zhang and Shen [8] studied estimation of a semi-parametric varying coefficient spatial panel data models. Dai et al. [9] investigated fixed effects quantile regression for general spatial panel data models with both individual fixed effect and time period effects based on instrumental variable method. Xu and Yang [10] proposed adjusted quasi score (AQS) tests for testing the existence of temporal heterogeneity in slope and spatial parameters in spatial panel data (SPD) models with fixed effects. Bai and Li [11] studied quasi-maximum likelihood estimator of dynamic spatial panel data models with common shocks to deal with both weak and strong cross-sectional correlations. Li and Yang [12] developed M-estimation and inference methods for spatial dynamic panel data models with correlated random effects based on short panels. Zhang et al. [13] studied a penalized quantile regression for spatial panel model with fixed effects. Except the work of [9, 13], all these works were developed based on (conditional) mean regression methods. Compared with mean regression methods, the quantile regression (QR) method is more robust and can be adopted to deal with data characterized by different error distributions.

However, in contrast to mean regression, there is no general transformation that can suitably eliminate the individual effects in the quantile regression framework (see, [14, 15]). Thus the FEQR estimation (see, [16]) is implemented by treating each individual effect also as a parameter to be estimated, which brings the computational difficulties. Hence, the IV-FEQR estimator (i.e., FEQR estimator based on instrumental variable method) used in [9] is also computational cumbersome. To address computational difficulties, [15] proposed the efficient minimum distance quantile regression (MDQR) method. Compared with the FEQR estimator, the MDQR estimator is computationally fast and is easy to implement in practice. The computing advantage is particularly obvious for large cross-sections.

In this paper, we employ the MDQR methodology for estimating the SAR panel data model with individual fixed effects. The instrumental variable (IV) method is employed to attenuate the estimation bias. The asymptotic properties of the IV-MDQR estimator are also developed. Monte Carlo simulations are conducted to assess the finite sample performance of the IV-MDQR, MDQR and IV-FEQR estimators. Computation speeds of IV-MDQR and IV-FEQR are also compared. Finally, We apply our theoretical results for the demand for cigarettes.

The rest of the paper is organized as follows. Section 2 introduces the SAR panel data model with individual fixed effects. Section 3 proposes the IV-MDQR estimation procedure. The asymptotic properties of the IV-MDQR estimators are also discussed. Proofs of the theorems in Sections 3 are given in the Appendix. Section 4 reports a simulation study for assessing the finite sample performance of the proposed estimators. An empirical illustration is considered in Section 5. Section 6 concludes the paper.

## 2 The model with individual fixed effects

Consider the following spatial autoregressive panel data (SARP) model with individual fixed effects:

$$\begin{array}{c} y_{it}=\rho \sum_{j=1}^{N}w_{ij}y_{jt}+X_{it}^{⊤}\beta +\eta_{i}+\varepsilon_{it}, \end{array}$$ (1)

where *y*_*it*_ is the dependent variable for subject *i* at time *t*, ***X***_*it*_ is a *p* × 1 vector of explanatory variables, *w*_*ij*_ is the (*i*, *j*)th element of ***W***, ***W*** is an *N* × *N* non-stochastic spatial weight matrix reflecting spatial dependence on *y*_*it*_ among cross sectional units, and *ε*_*it*_ is the random disturbance term. The parameters *η*_*i*_, *i* = 1, ⋯, *N* are fixed effects for the regions. Interaction effects are reflected in the spatial lag variable $\sum_{j=1}^{N}w_{ij}y_{jt}$ (and associated scalar parameter *ρ*).

We consider the following conditional *τ*-quantile of response variable:

$$\begin{array}{c} Q_{\tau }\left(y_{it}|D_{it},X_{it},Z_{i}\right)=\rho \left(\tau \right)D_{it}+X_{it}^{⊤}\beta \left(\tau \right)+Z_{i}^{⊤}\eta \left(\tau \right), \end{array}$$ (2)

where *τ* is a quantile in the interval (0, 1), *Q*_*τ*_(*ε*_*it*_|*D*_*it*_, ***X***_*it*_, ***Z***_*i*_) = 0, $D_{it}=\sum_{j=1}^{N}w_{ij}y_{jt}$, $\eta ={\left(\eta_{1},\cdots ,\eta_{N}\right)}^{⊤}$, ***Z***_*i*_ = ***Z***^⊤^ ***h***_*i*_ is an indicator variable for the individual effect *η*_*i*_, ***h***_*i*_ is an *NT* × 1 vector with the *i*th element equal to 1 and the rest equal to 0, ***Z*** = **1**_*T*_ ⊗ **I**_*N*_ is an *NT* × *N* matrix, **1**_*T*_ is the *T* × 1 vector with all the elements being 1.

And the FEQR estimator can then be obtained by minimizing the following objection function:

$$\begin{array}{c} \left(\hat{\rho }\left(\tau \right),\hat{\beta }\left(\tau \right),\hat{\eta }\left(\tau \right)\right)=\underset{\rho ,\beta ,\eta }{\text{arg}\;\text{min}}\sum_{i=1}^{N}\sum_{t=1}^{T}\rho_{\tau }\left(y_{it}-\rho \left(\tau \right)D_{it}-X_{it}^{⊤}\beta \left(\tau \right)-Z_{i}^{⊤}\eta \left(\tau \right)\right), \end{array}$$ (3)

where *ρ*_*τ*_(*u*) = *u*(*τ* − *I*(*u* ≤ 0)) is the check function and *I*(⋅) is the indicator function (see, e.g., [17]).

Galvao and Wang [15] argued that unlike mean regression, the individual effects cannot be suitably eliminated via transformation in the FEQR estimator. Thus the FEQR estimator is implemented by treating each individual effect *η*_*i*_ as a parameter to be estimated. Therefore, if the number of the individuals is large, the FEQR estimator will involve optimization with large number of parameters to be estimated, which makes the problem computationally cumbersome. Inference using the FEQR estimator is difficult to conduct in practice. For this reason, we employ the minimum distance quantile regression (MDQR) estimator (see, [15]) for estimation.

Denote ***θ*** = (*ρ*, ***β***^⊤^)^⊤^. The MDQR estimation of model (1) can be implemented via the following two steps:

Step 1: Obtain the QR estimation ${\hat{\theta }}_{i}$ and ${\hat{\eta }}_{i}$ using the time series data of each individual *i*. Denote **V**_*i*_ the associated variance-covariance matrix of ${\hat{\theta }}_{i}$ for each individual, i.e., $V_{i}=\tau \left(1-\tau \right)E{\left(f_{i}\left(0|{\tilde{X}}_{it}\right){\tilde{X}}_{it}{\tilde{X}}_{it}^{⊤}\right)}^{-1}E\left({\tilde{X}}_{it}{\tilde{X}}_{it}^{⊤}\right)E{\left(f_{i}\left(0|{\tilde{X}}_{it}\right){\tilde{X}}_{it}{\tilde{X}}_{it}^{⊤}\right)}^{-1}$, where ${\tilde{X}}_{it}={\left[D_{it},X_{it}^{⊤}\right]}^{⊤}$, $f_{i}\left(0|{\tilde{X}}_{it}\right)$ is the conditional density of *ε*_*it*_ at the quantile of interest.
Step 2: Then the MDQR estimator can be defined by
  $$\hat{\theta }={\left(\sum_{i=1}^{N}{\hat{V}}_{i}^{-1}\right)}^{-1}\sum_{i=1}^{N}{\hat{V}}_{i}^{-1}{\hat{\theta }}_{i},$$
  where ${\hat{V}}_{i}$ is the estimator of **V**_*i*_.

## 3 The IV-MDQR estimator

However, there exist an endogenous variable in model (2), i.e., the spatial lag *D*_*it*_, which can cause biased estimation. Thus the MDQR estimation of model (2) is biased especially for the spatial correlation coefficient *ρ*. The problem of bias for quantile regression for spatial autoregressive panel data model can be ameliorated through the use of instrumental variables. Therefore, we employ the instrumental variable method for bias reduction in this section.

Suppose the endogenous variable *D*_*it*_ is related to a vector of instruments ***ω***_*it*_, and the instruments ***ω***_*it*_ are independent of *ε*_*it*_. Following [18–20], and assuming the availability of instrumental variables ***ω***_*it*_, we can derive the IV-MDQR estimator via the following four steps:

Step 1: For each individual *i* and a given quantile *τ*, define a suitable set of values {*ρ*_*j*_, *j* = 1, ⋯, *J*;|*ρ*| < 1}. One can obtain the ordinary QR estimation ${\hat{\beta }}_{i},\eta_{i},{\hat{\gamma }}_{i}$ of each individual *i* using the time series data via minimizing the following objective function:
  $$\begin{array}{c} R_{i}^{IV}\left(\tau ,\rho ,\beta ,\eta_{i},\gamma \right)=\sum_{t=1}^{T}\rho_{\tau }\left(y_{it}-\rho D_{it}-X_{it}^{⊤}\beta -\eta_{i}-\omega_{it}^{⊤}\gamma \right), \end{array}$$ (4)
  where *γ* is the coefficient of the instrumental variable ***ω***_*it*_.
Step 2: Choose $\hat{\rho }\left(\tau \right)$ among {*ρ*_*j*_, *j* = 1, ⋯, *J*} which makes a weighted distance function defined on ***γ*** closest to 0:
  $$\begin{array}{c} {\hat{\rho }}_{i}\left(\tau \right)=\underset{\rho \in R}{\text{arg}\;\text{min}}\left|{\hat{\gamma }}_{i}\left(\rho ,\tau \right)\right|, \end{array}$$ (5)
  where R is the parameter space of *ρ*.
Step 3: The IVQR estimation of $\beta ,\eta_{i}$ can be obtained, which is respectively ${\hat{\beta }}_{i}\left({\hat{\rho }}_{i}\left(\tau \right),\tau \right)$, ${\hat{\eta }}_{i}\left({\hat{\rho }}_{i}\left(\tau \right),\tau \right)$. Therefore, ${\hat{\theta }}_{i}={\left({\hat{\rho }}_{i}\left(\tau \right),{\hat{\beta }}_{i}{\left({\hat{\rho }}_{i}\left(\tau \right),\tau \right)}^{⊤}\right)}^{⊤}$.
Step 4: Then the IV-MDQR estimator of SAR panel data model (1) can be defined by
  $$\begin{array}{c} \hat{\theta }={\left(\sum_{i=1}^{N}{\hat{\tilde{V}}}_{i}^{-1}\right)}^{-1}\sum_{i=1}^{N}{\hat{\tilde{V}}}_{i}^{-1}{\hat{\theta }}_{i}, \end{array}$$ (6)
  where ${\hat{\tilde{V}}}_{i}$ is the estimation of ${\tilde{V}}_{i}$, ${\tilde{V}}_{i}$ the associated variance-covariance matrix of the IVQR estimator ${\hat{\theta }}_{i}$ for each individual *i*, which takes the form:
  $${\tilde{V}}_{i}=J_{i}{\left(\tau \right)}^{-1}S_{i}\left(\tau \right){\left[J_{i}{\left(\tau \right)}^{-1}\right]}^{⊤},$$
  where $J_{i}\left(\tau \right)=E\left(f_{i}\left(0|D_{it},X_{it},\omega_{it}\right)X_{it}^{*}{\tilde{X}}_{it}^{⊤}\right)$, $S_{i}\left(\tau \right)=\tau \left(1-\tau \right)E\left(X_{it}^{*}X_{it}^{*⊤}\right)$, $X_{it}^{*}={\left[\omega_{it}^{⊤},X_{it}^{⊤}\right]}^{⊤}$, ${\tilde{X}}_{it}={\left[D_{it},X_{it}^{⊤}\right]}^{⊤}$, $f_{i}\left(0|D_{it},X_{it},\omega_{it}\right)$ is the conditional density of *ε*_*it*_ at the quantile of interest.

**Remark 3.1**. For each individual *i*, we need instruments for the endogenous variables $D_{it}=w_{i}^{⊤}y_{t}$, where ***W***_*i*_ is the *i*th row of the spatial weight matrix ***W***. The instruments need to satisfy the following two conditions: (i) instruments ***ω***_*it*_ can impact the endogenous variables *D*_*it*_; (ii) instruments ***ω***_*it*_ are independent of the random error *ε*_*it*_. In practice, for spatial autoregressive panel data model (1), we can choose the time-lag of *y*_*it*_, i.e., *y*_*it*−1_ and the spatial lag of the explanatory variable, i.e., $w_{i}^{⊤}X_{t}$, as instrumental variable.

### 3.1 Asymptotic theory

In this section, we investigate the asymptotic properties of the IV-MDQR estimator. We impose the following regularity conditions:

**A1** {(*y*_*it*_, *X*_*it*_)} is independent across individuals, and is independent and identically distributed (i.i.d.) within each *i*.
**A2** For all τ∈T, (ρ(τ),β(τ),η(τ)) is in the interior of the set R×B×E, and R×B×E is compact and convex.
**A3** $\text{max}\|y_{it}\|=O\left(\sqrt{NT}\right)$ , $\text{max}\|X_{it}\|=O\left(\sqrt{NT}\right)$ and $\text{max}\|\omega_{it}\|=O\left(\sqrt{NT}\right)$.
**A4** ***W*** is non-stochastic spatial weights matrices with zero diagonals. ***W*** is uniformly bounded in both row and column sums in absolute value.
**A5** For each individual *i*, for
  $$\begin{array}{c} \Pi (\rho ,\beta ,\eta_{i},\tau )=E\left[\left(\tau -I\left(y_{i}<\rho D_{i}+X_{i}\beta +\eta_{i}\right)\right)\right)\Delta_{i}], \end{array}$$ (7)
  $$\begin{array}{c} \Pi (\rho ,\beta ,\eta_{i},\gamma ,\tau )=E[\left(\tau -I\left(y_{i}<\rho D_{i}+X_{i}\beta +\eta_{i}+\omega_{i}\gamma \right)\right)\Delta_{i}], \end{array}$$ (8)
  where **Δ**_*i*_ = [***ω***_*i*_, ***X***_*i*_, **1**_*T*_], ***X***_*i*_ = (***X***_*i*1_, ⋯, ***X***_*iT*_)^⊤^, **ω**_*i*_ = (***ω***_*i*1_, ⋯, ***ω***_*iT*_)^⊤^. The Jacobian matrices $\frac{\partial \Pi (\rho ,\beta ,\eta_{i},\tau )}{\partial (\rho ,\beta ,\eta_{i})}$ and $\frac{\partial \Pi (\rho ,\beta ,\eta_{i},\gamma ,\tau )}{\partial (\beta ,\eta_{i},\gamma )}$ are continuous and have full rank uniformly over B×E×G×T. The parameter space R×B×E is a connected set and the image of R×B×E under the map $\left(\rho ,\beta ,\eta_{i}\right)\mapsto \Pi \left(\rho ,\beta ,\eta_{i},\tau \right)$ is simply connected.
**A6** The conditional density *f*_*i*_(*ε*|***D***, ***X***) is continuously differentiable for each *i*. There exist 0 < *C*_*L*_ ≤ *C*_*U*_ < ∞ such that *f*_*i*_(*ε*|***D***, ***X***)≤*C*_*U*_ uniformly over $\left(\varepsilon ,\tilde{X}\right)$ and *i* ≥ 1, and *f*_*i*_(0|***D***, ***X***)≥*C*_*L*_ uniformly over $\tilde{X}$ and *i* ≥ 1; and there exists *C*_*f*_ > 0 such that $|f_{i}^{\prime }\left(\varepsilon |D,X\right)|\leq C_{f}$. Here, $\tilde{X}=\left[D,X\right]$, $f_{i}^{\prime }\left(\varepsilon |D,X\right)$ is the first-order derivative of the density *f*_*i*_(*ε*|***D***, ***X***).
**A7** There exists *δ*_*S*_ > 0 such that min_1≤*i* ≤ *N*_ min eig(***S***_*i*_(*τ*)) ≥ *δ*_*S*_, where $S_{i}\left(\tau \right)=\tau \left(1-\tau \right)E\left(X_{it}^{*}X_{it}^{*⊤}\right)$, $X_{it}^{*}={\left[\omega_{it}^{⊤},X_{it}^{⊤}\right]}^{⊤}$.

Assumptions A1-A3 and A6 are standard in the literature on quantile regression for panel data. Assumption A4 is originated by [21, 22] and is also used in [7, 9]. Assumption A5 is a standard assumption in the instrumental variable quantile regression literature. Assumption A7 assures that $S_{i}^{-1}\left(\tau \right)$ are bounded uniformly across *i*. Assumptions A3 and A7 guarantee that both $J_{i}\left(\tau \right)=E\left(f_{i}\left(0|D_{it},X_{it},\omega_{it}\right)X_{it}^{*}{\tilde{X}}_{it}^{⊤}\right)$ and their inverses are bounded uniformly across *i*.

In applications, the variance-covariance matrices are unknown and need to be estimated. Following [15], when *T* and *N* tend to infinity sequentially, we impose the following assumption:

**A8** ${\hat{\tilde{V}}}_{i}={\tilde{V}}_{i}+o_{p}\left(1\right)$ for each *i* as *T* → ∞. Assume that ${\left(N^{-1}\sum_{i=1}^{N}{\tilde{V}}_{i}^{-1}\right)}^{-1}\to \tilde{V}$, where $\tilde{V}={\text{lim}}_{N\to \infty }{\left(\frac{1}{N}\sum_{i=1}^{N}{\tilde{V}}_{i}^{-1}\right)}^{-1}$ exists and is non-singular.
  When *N* and *T* tend to infinity simultaneously, we make the following assumption:
**A8′** ${\hat{\tilde{V}}}_{i}={\tilde{V}}_{i}+O_{p}\left(T^{-1/2}h_{N}^{-1/2}\right)$ for some *h*_*N*_ → 0 uniformly across *i* and ${\text{lim}}_{N\to \infty }\frac{N}{Th_{N}}=0$ as *N* → ∞. Assume that ${\left(N^{-1}\sum_{i=1}^{N}{\tilde{V}}_{i}^{-1}\right)}^{-1}\to \tilde{V}$, where $\tilde{V}={\text{lim}}_{N\to \infty }{\left(\frac{1}{N}\sum_{i=1}^{N}{\tilde{V}}_{i}^{-1}\right)}^{-1}$ exists and is non-singular.

We can now establish consistency and asymptotic normality of the IV-MDQR estimator. Proofs are given in the Appendix.

**Theorem 3.1**. *1. Under assumptions A1-A6 and A8*, (*ρ*(*τ*), ***β***(*τ*)) *is consistently estimable as* (*T*, *N*)_*seq*_ → ∞.

*2. Under assumptions A1-A6 and A8′*, (*ρ*(*τ*), ***β***(*τ*)) *is consistently estimable as* (*T*, *N*)→∞ *and* log*N*/*T* → 0.

**Theorem 3.2**. *1. Under assumptions A1-A7 and A8, as* (*T*, *N*)_*seq*_ → ∞,

$$\sqrt{NT}\left(\hat{\theta }\left(\tau \right)-\theta \left(\tau \right)\right)\overset{d}{\to }N\left(0,\tilde{V}\right).$$

*2. Under assumptions A1-A7 and A8′, as* (*T*, *N*) → ∞ *and* $\frac{N^{2}\text{log}N}{T}|\text{log}\frac{{\left(\text{log}N\right)}^{1/2}}{T^{1/2}}{|}^{2}\to 0$,

$$\sqrt{NT}\left(\hat{\theta }\left(\tau \right)-\theta \left(\tau \right)\right)\overset{d}{\to }N\left(0,\tilde{V}\right).$$

## 4 Monte Carlo simulations

In this section, we conduct Monte Carlo simulations to investigate the finite sample performance of the IV-MDQR estimator. We report results for average bias and root mean squared error (RMSE). We are mainly interested in comparing the performances of the IV-MDQR estimator and other two QR estimators, such as MDQR and IV-FEQR. The Monte Carlo simulations are repeated 1000 times. We consider several sample sizes and quantiles, where *N* ∈ {50, 100, 200}, *T* ∈ {50, 100}, and *τ* ∈ {0.25, 0.5, 0.75}. For IV-MDQR and IV-FEQR, we search *ρ* from {0.05k:-20≤k≤20,k∈Z}.

The samples are generated as follows:

**Example 1. (homoscedastic case)**

$$y_{it}=\rho \sum_{i=1}^{N}w_{ij}y_{jt}+X_{it}\beta +\eta_{i}+\varepsilon_{it}.$$

**Example 2. (heteroscedastic case)**

$$y_{it}=\rho \sum_{i=1}^{N}w_{ij}y_{jt}+X_{it}\beta +\eta_{i}+\left(1+0.1X_{it}\right)\varepsilon_{it}.$$

In the two cases, we set *ρ* = 0.5, *β* = 1. And ***X***, ***η*** are drawn respectively from *U*(−2, 2) and *N*(0, 1) distribution. The spatial weights matrix ***W*** is generated based on the mechanism considered in Zhang and Shen (2015), i.e., $W=I\left(N/10\right)⊗\left(\frac{1_{10}1_{10}^{⊤}-I_{10}}{9}\right)$. *ε*_*it*_ = *e*_*it*_ − *F*^−1^(*τ*), *F* is the common CDF of *e*_*it*_. Therefore, the random errors *ε*_*it*_ are centered to have zero *τ*th quantile. For the disturbance errors, we consider the standard normal (i.e., *N*(0, 1)) distribution.

Tables 1 and 2 respectively reports the bias and RMSE of the several QR estimators in the homoscedastic and heteroscedastic case. For IV-MDQR and IV-FEQR, we considered two different instruments, *y*_*it*−1_ and the spatial lag of ***X***_*it*_. The results are similar in both cases, and we simply present results for the *y*_*it*−1_ case. From Tables 1 and 2, we see that the bias and RMSE of the estimators are obviously reduced as the sample size increase except the MDQR estimator. The IV-MDQR overwhelmingly performs better than the MDQR estimator, which shows that the instrumental variable method effectively reduces the estimation bias. For estimating the coefficient *β*, the IV-MDQR and the IV-FEQR estimator perform similarly. For estimating the spatial correlation coefficient *ρ*, the IV-MDQR estimator has larger bias but smaller RMSE than the IV-FEQR estimator.

**Table 1:** Bias and RMSE (in parentheses) of various estimators in the homoscedastic case.

*T*	*N*	Para.	*τ* = 0.25			*τ* = 0.5			*τ* = 0.75		
			IVMD	MD	IVFE	IVMD	MD	IVFE	IVMD	MD	IVFE
50	50	*ρ*	0.013	0.108	-0.009	0.012	0.109	-0.008	0.016	0.110	-0.004
			(0.029)	(0.112)	(0.061)	(0.026)	(0.112)	(0.060)	(0.029)	(0.114)	(0.063)
		*β*	-0.003	-0.016	0.001	-0.002	-0.010	-0.000	-0.001	-0.015	0.001
			(0.023)	(0.031)	(0.033)	(0.022)	(0.022)	(0.033)	(0.024)	(0.028)	(0.032)
	100	*ρ*	0.012	0.108	-0.003	0.010	0.108	-0.003	0.014	0.111	-0.002
			(0.023)	(0.110)	(0.040)	(0.020)	(0.110)	(0.037)	(0.024)	(0.113)	(0.040)
		*β*	-0.001	-0.010	0.001	0.000	-0.011	-0.000	-0.002	-0.014	0.001
			(0.018)	(0.020)	(0.024)	(0.016)	(0.020)	(0.022)	(0.018)	(0.023)	(0.022)
	200	*ρ*	0.011	0.112	0.000	0.009	0.111	0.000	0.013	0.112	0.000
			(0.019)	(0.113)	(0.029)	(0.019)	(0.112)	(0.027)	(0.020)	(0.113)	(0.030)
		*β*	-0.001	-0.012	0.001	-0.001	-0.012	0.001	-0.000	-0.012	0.001
			(0.012)	(0.017)	(0.018)	(0.010)	(0.016)	(0.015)	(0.013)	(0.018)	(0.016)
100	50	*ρ*	0.011	0.112	-0.004	0.011	0.111	-0.001	0.010	0.111	0.000
			(0.022)	(0.113)	(0.041)	(0.022)	(0.112)	(0.038)	(0.021)	(0.113)	(0.048)
		*β*	-0.002	-0.008	-0.001	-0.002	-0.012	-0.004	-0.001	-0.011	-0.001
			(0.017)	(0.019)	(0.025)	(0.016)	(0.021)	(0.023)	(0.016)	(0.020)	(0.024)
	100	*ρ*	0.010	0.112	-0.001	0.010	0.112	-0.002	0.010	0.110	-0.001
			(0.018)	(0.113)	(0.029)	(0.016)	(0.113)	(0.031)	(0.018)	(0.111)	(0.029)
		*β*	-0.000	-0.011	-0.002	-0.000	-0.011	0.000	-0.001	-0.012	0.000
			(0.013)	(0.015)	(0.018)	(0.011)	(0.015)	(0.015)	(0.012)	(0.017)	(0.019)
	200	*ρ*	0.010	0.112	-0.001	0.009	0.111	0.001	0.011	0.112	-0.001
			(0.014)	(0.112)	(0.023)	(0.014)	(0.111)	(0.020)	(0.016)	(0.112)	(0.021)
		*β*	-0.001	-0.010	-0.001	-0.001	-0.010	0.001	-0.001	-0.012	0.000
			(0.009)	(0.013)	(0.011)	(0.007)	(0.013)	(0.011)	(0.009)	(0.015)	(0.011)

**Table 2:** Bias and RMSE (in parentheses) of various estimators in the heteroscedastic case.

*T*	*N*	Para.	*τ* = 0.25			*τ* = 0.5			*τ* = 0.75		
			IVMD	MD	IVFE	IVMD	MD	IVFE	IVMD	MD	IVFE
50	50	*ρ*	0.012	0.100	0.002	0.014	0.107	-0.003	0.015	0.116	-0.006
			(0.027)	(0.104)	(0.043)	(0.027)	(0.110)	(0.040)	(0.028)	(0.120)	(0.044)
		*β*	0.002	-0.010	-0.001	-0.003	-0.013	0.001	-0.004	-0.016	-0.003
			(0.024)	(0.025)	(0.023)	(0.021)	(0.027)	(0.023)	(0.023)	(0.029)	(0.023)
	100	*ρ*	0.011	0.102	-0.002	0.014	0.107	-0.002	0.014	0.118	0.001
			(0.022)	(0.104)	(0.029)	(0.021)	(0.109)	(0.028)	(0.021)	(0.120)	(0.032)
		*β*	0.000	-0.008	-0.002	0.000	-0.008	0.000	-0.005	-0.015	-0.001
			(0.018)	(0.019)	(0.016)	(0.017)	(0.019)	(0.015)	(0.018)	(0.024)	(0.017)
	200	*ρ*	0.010	0.100	0.001	0.012	0.108	-0.002	0.013	0.116	-0.001
			(0.019)	(0.101)	(0.018)	(0.013)	(0.108)	(0.021)	(0.020)	(0.117)	(0.020)
		*β*	0.001	-0.006	0.000	-0.002	-0.012	0.000	-0.006	-0.015	0.001
			(0.012)	(0.014)	(0.016)	(0.011)	(0.016)	(0.015)	(0.014)	(0.019)	(0.016)
100	50	*ρ*	0.009	0.101	0.002	0.009	0.109	0.001	0.008	0.118	-0.002
			(0.021)	(0.103)	(0.032)	(0.021)	(0.111)	(0.028)	(0.022)	(0.120)	(0.035)
		*β*	0.001	-0.008	0.000	-0.002	-0.011	0.000	-0.002	-0.015	-0.002
			(0.017)	(0.019)	(0.018)	(0.016)	(0.019)	(0.015)	(0.016)	(0.023)	(0.016)
	100	*ρ*	0.008	0.100	-0.001	0.008	0.109	0.000	0.007	0.118	0.001
			(0.016)	(0.101)	(0.021)	(0.016)	(0.110)	(0.017)	(0.018)	(0.119)	(0.025)
		*β*	0.001	-0.007	0.000	-0.001	-0.011	-0.000	-0.002	-0.013	-0.000
			(0.012)	(0.013)	(0.012)	(0.012)	(0.015)	(0.010)	(0.013)	(0.017)	(0.012)
	200	*ρ*	0.007	0.102	-0.000	0.006	0.110	-0.000	0.007	0.118	-0.000
			(0.013)	(0.102)	(0.013)	(0.013)	(0.110)	(0.009)	(0.015)	(0.118)	(0.013)
		*β*	0.000	-0.010	0.000	-0.001	-0.011	0.000	-0.002	-0.013	-0.000
			(0.008)	(0.013)	(0.008)	(0.008)	(0.014)	(0.008)	(0.009)	(0.016)	(0.008)

Following we compare the computing time of the IV-MDQR and IV-FEQR at one particular quantile *τ* = 0.5 in Example 1. We are interest in the elapsed time, i.e., the time required for one replication of simulation. The results are summarized in the following Table 3. Table 3 shows that as the sample size increase, the computing times of both the IV-MDQR and the IV-FEQR estimators increase, but the increase rate of IV-FEQR estimator is much faster than the IV-MDQR estimator.

**Table 3:** The computing time (in seconds) of IV-FEQR and IV-MDQR estimators required for one replication under different sample size.

	IV-MDQR	IV-FEQR
*N* = *T* = 10	0.200	0.292
*N* = *T* = 50	1.425	0.609
*N* = *T* = 100	3.463	10.395
*N* = *T* = 250	13.372	547.868
*N* = *T* = 500	39.590	30025.183

Moreover, we are also interested in the question whether the computing time of the estimators is more sensitive to *T* and *N*. We consider the following two situations: (1) fix *T* = 100 and *N* varies in {10, 50, 100, 250, 500}; (2) fix *N* = 100 and *T* varies in {10, 50, 100, 250, 500}. The results are summarized in the following Table 4. From which, we can see that the computing time of the two estimators both are much more sensitive to the the size of *N*. But the sensitivity of IV-MDQR estimator is much lower than the IV-FEQR estimator.

**Table 4:** The computing time (in seconds) of IV-FEQR and IV-MDQR estimators required for one replication under different sample size.

*N*	*T* = 100		*T*	*N* = 100	
	IV-MDQR	IV-FEQR		IV-MDQR	IV-FEQR
10	0.351	0.169	10	1.927	1.781
50	1.728	2.238	50	2.925	4.614
100	3.463	10.395	100	3.463	10.395
250	8.692	117.500	250	5.619	64.759
500	17.730	5469.055	500	8.099	381.982

## 5 Illustration

In this section, we employ the cigarette demand data set (https://spatial-panels.com/software/) to illustrate our methodologies. The cigarette demand data set has been analyzed by many authors (see, [9, 23–27]). The data set is based on a panel of 46 states and covers 1963 to 1992. The spatial weight matrix ***W*** is also given in the data set. We take the following two variables as explanatory variables, such as the logarithm average retail price of a pack of cigarettes measured in real terms, (*X*_1_), and the logarithm real per capita disposable income (*X*_2_). The dependent variable *y*_*it*_ is the logarithm real per capita sales of cigarettes by persons of smoking age (14 years and older).

**Fig 1:**
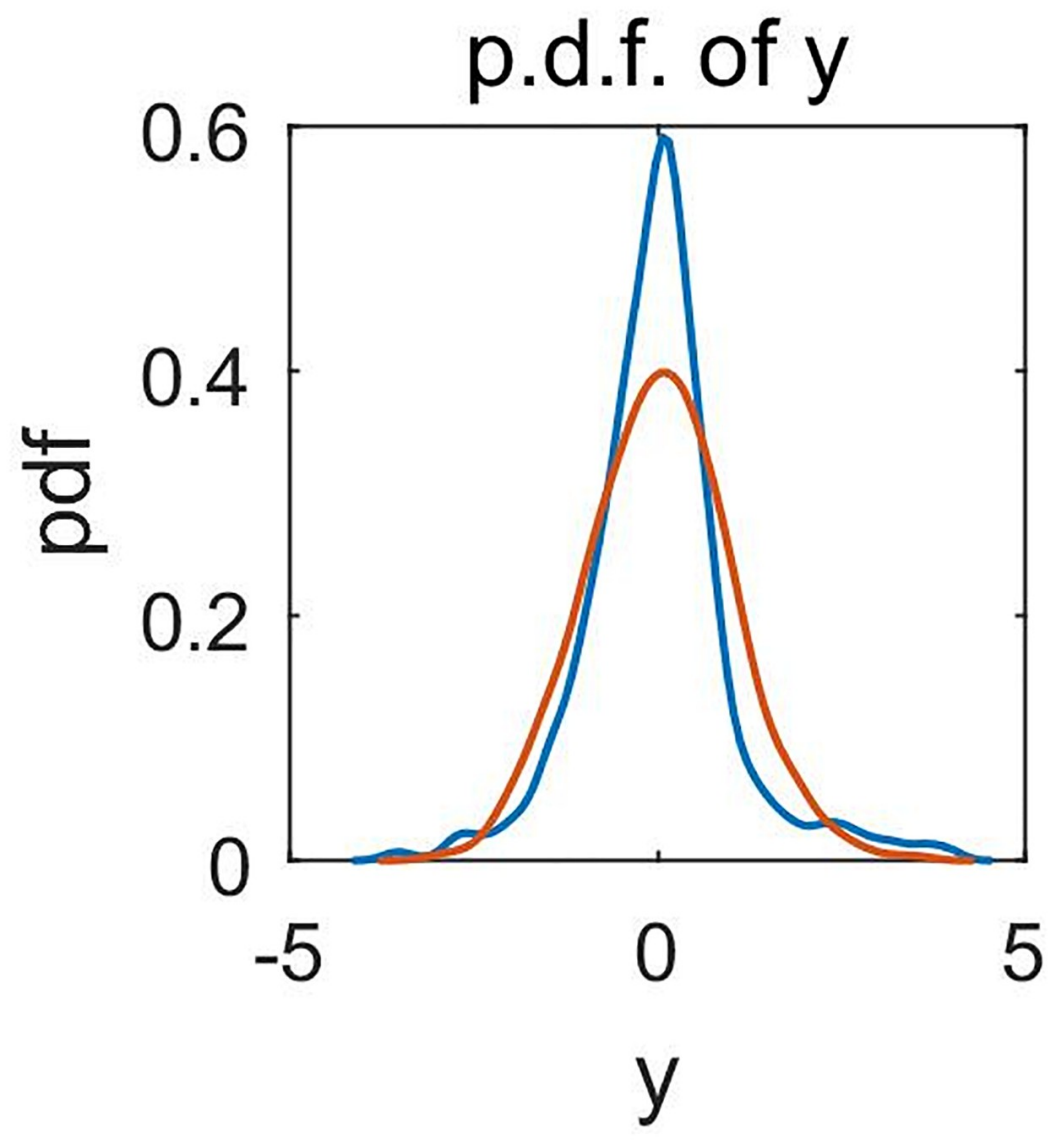
Probability density plots of *y* in the cigarette demand data set (blue line) and *N*(0, 1) (orange line).

Firstly, the Kolmogorov-Smirnov test is employed to test whether the standardized **y** follows the standard normal distribution. The result shows that the normality assumption is rejected at the 0.05 significance level. Fig 1 gives the p.d.f. plot of response *y*, which shows that the density of *y* has larger kurtosis than *N*(0, 1).

Following, we employ the spatial autoregressive panel data model for analysis. The fitted model takes the form:

$$\begin{array}{c} Q_{\tau }\left(y_{it}|D_{it},X_{it}\right)=\rho \left(\tau \right)D_{it}+X_{1,it}\beta_{1}\left(\tau \right)+X_{2,it}\beta_{2}\left(\tau \right)+Z_{1i}^{⊤}\eta \left(\tau \right), \end{array}$$ (9)

where $D_{it}=\sum_{j=1}^{N}w_{ij}y_{jt}$.

We estimate the parameters using the IV-MDQR, IV-FEQR, MLE, and OLS methods. The results are presented in Table 5. The first three columns are the IV-MDQR estimates for *τ* = 0.25, 0.5, 0.75, the middle three columns are the IV-FEQR estimates for *τ* = 0.25, 0.5, 0.75, and the last two columns correspond to the MLE and OLS estimates respectively. We can see that the IV-MDQR and IV-FEQR estimates both vary at different quantiles (i.e., *τ* = 0.25, 0.5, 0.75). Except *β*_2_, the signs of the estimates are the same among IV-MDQR, IV-FEQR, MLE and OLS methods. At quantiles 0.25, 0.5 and 0.75, the cigarettes sales between neighbour states has a positive effect to each other, the log average cigarettes retail price has a negative effect to the cigarettes sales, and the log disposable income generally has a positive effect to the cigarettes sales.

**Table 5:** Estimation results of cigarette demand based on spatial autoregressive panel data models.

Para.	IV-MDQR			IV-MDQR			MLE	OLS
	*τ* = 0.25	*τ* = 0.50	*τ* = 0.75	*τ* = 0.25	*τ* = 0.50	*τ* = 0.75		
*ρ*	0.431	0.345	0.402	0.610	0.490	0.410	0.286	0.424
*β*_1_	-0.535	-0.565	-0.601	-0.343	-0.348	-0.350	-0.530	-0.451
*β*_2_	0.104	0.029	0.075	0.114	0.035	-0.016	-0.003	0.002

**Fig 2:**
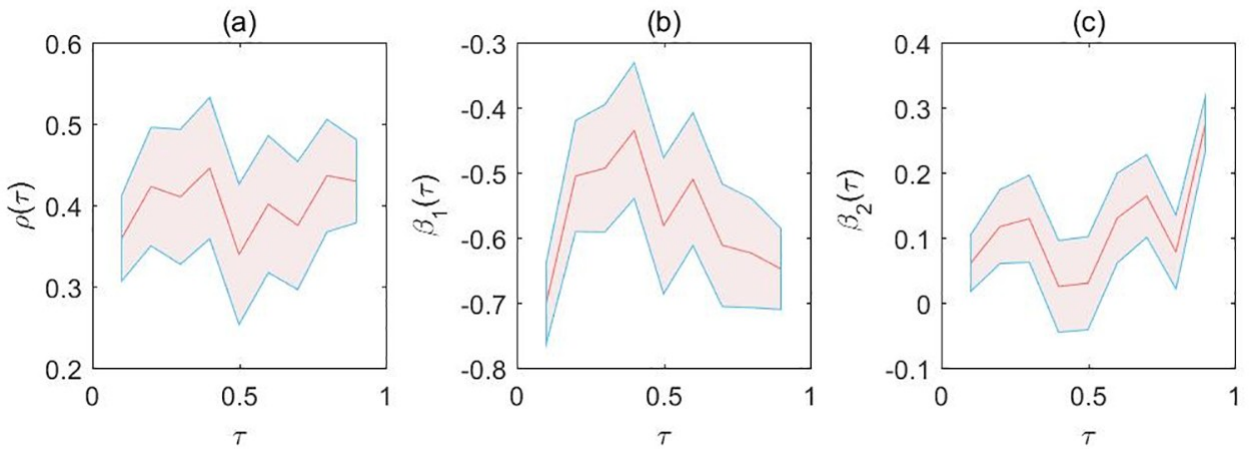
Quantile effects of the spatial correlation coefficient, the log average retail price of a pack of cigarettes and the log disposable income. The areas represent 95% point-wise confidence intervals.

Fig 2 presents a complete analysis, which considers other quantiles of the conditional cigarettes demand distribution. The *x*-axis presents the quantiles and *y*-axis presents the IV-MDQR estimations of parameters (red lines) and their corresponding confidence intervals (blue lines). We find that the cigarettes retail price has negative effect to the capita sales of cigarettes and disposable income has positive effect to the capita sales of cigarettes at all quantiles levels. Besides, the effects of capita sales of cigarettes and disposable income are both larger at extreme quantiles.

## 6 Conclusion

In this paper, we investigate minimum distance quantile regression (IV-MDQR) estimation of spatial autoregressive panel data models with fixed individual effects. The instrumental variable method is employed for bias reduction. The asymptotic properties are studied. Monte Carlo results are provided to show that the proposed methodology effectively reduces the estimation bias and is computationally advantageous.

## 7 Appendix: Proofs

### 7.1 Proof of Theorem 3.1

*Proof*. Denote $\vartheta_{i0}={\left(\theta_{0}^{⊤},\eta_{i0}\right)}^{⊤}={\left(\rho_{0},\beta_{0}^{⊤},\eta_{i0}\right)}^{⊤}$. Firstly, ${\hat{\vartheta }}_{i}\left(\tau \right)$ is the IVQR estimator which is computed using the time series data. Following [18], under assumptions A1-A6, the IVQR estimation of $\left({\hat{\rho }}_{i}\left(\tau \right),{\hat{\beta }}_{i}\left(\tau \right),{\hat{\eta }}_{i}\left(\tau \right)\right)\overset{p}{\to }\left(\rho_{0}\left(\tau \right),\beta_{0}\left(\tau \right),\eta_{i0}\left(\tau \right)\right)$ as *T* → ∞ for each individual *i*.

1. By assumption A8, ${\hat{\tilde{V}}}_{i}\overset{p}{\to }{\tilde{V}}_{i}$ for each *i*, it follows that for fixed *N*, as *T* → ∞
  $$\hat{\theta }\left(\tau \right)={\left(\sum_{i=1}^{N}{\hat{\tilde{V}}}_{i}^{-1}\right)}^{-1}\sum_{i=1}^{N}{\hat{\tilde{V}}}_{i}^{-1}{\hat{\theta }}_{i}\left(\tau \right)\overset{p}{\to }{\left(\sum_{i=1}^{N}{\tilde{V}}_{i}^{-1}\right)}^{-1}\sum_{i=1}^{N}{\tilde{V}}_{i}^{-1}\theta_{0}\left(\tau \right)=\theta_{0}\left(\tau \right).$$
  Hence, it follows that $\left(\hat{\rho }\left(\tau \right),\hat{\beta }\right)\overset{p}{\to }\left(\rho_{0}\left(\tau \right),\beta_{0}\left(\tau \right)\right)$ as (*T*, *N*)_seq_ → ∞.
2. To show the consistency of $\hat{\theta }$ for joint asymptotics, we do the following computation:
  $$\begin{array}{cc} \hat{\theta }\left(\tau \right)-\theta_{0}\left(\tau \right) & ={\left(\sum_{i=1}^{N}{\hat{\tilde{V}}}_{i}^{-1}\right)}^{-1}\sum_{i=1}^{N}{\hat{\tilde{V}}}_{i}^{-1}\left({\hat{\theta }}_{i}\left(\tau \right)-\theta_{0}\left(\tau \right)\right)\\ & ={\left(\sum_{i=1}^{N}{\hat{\tilde{V}}}_{i}^{-1}\right)}^{-1}\sum_{i=1}^{N}{\hat{\tilde{V}}}_{i}^{-1}o_{p}\left(1\right). \end{array}$$
  Under assumptions A1-A5, based on Lemma 1 in [15], we have ${\text{max}}_{1\leq i\leq N}\left\|{\hat{\theta }}_{i0}\left(\tau \right)-\theta_{0}\left(\tau \right)\right\|=o_{p}\left(1\right)$ as (*N*, *T*)→∞ and $\frac{\text{log}\;N}{T}\to 0$. Hence, the last equation above is equal to *o*_*p*_(1).

### 7.2 Proof of Theorem 3.2

*Proof*. We first derive the asymptotic normality of the IV-MDQR estimator under sequential asymptotics. Under Assumptions A1-A6, for each individual, the IVQR estimator ${\hat{\theta }}_{i}\left(\tau \right)$ converges to a Gaussian distribution:

$$\sqrt{T}\left({\hat{\theta }}_{i}\left(\tau \right)-\theta_{0}\left(\tau \right)\right)\overset{d}{\to }N\left(0,{\tilde{V}}_{i}\right),$$

where ${\tilde{V}}_{i}=J_{i}{\left(\tau \right)}^{-1}S_{i}\left(\tau \right){\left[J_{i}{\left(\tau \right)}^{-1}\right]}^{⊤}$, $J_{i}\left(\tau \right)=E\left(f_{i}\left(0|D_{it},X_{it},\omega_{it}\right)X_{it}^{*}{\tilde{X}}_{it}^{⊤}\right)$, $S_{i}\left(\tau \right)=\tau \left(1-\tau \right)E\left(X_{it}^{*}X_{it}^{*⊤}\right)$, $X_{it}^{*}={\left[\omega_{it}^{⊤},X_{it}^{⊤}\right]}^{⊤}$, ${\tilde{X}}_{it}={\left[D_{it},X_{it}^{⊤}\right]}^{⊤}$, $f_{i}\left(0|D_{it},X_{it},\omega_{it}\right)$ is the conditional density of *ε*_*it*_ at the quantile of interest (see, [18, 19]).

By assumption A8 and Slutsky’s theorem, $\sqrt{T}\left({\hat{\theta }}_{i}\left(\tau \right)-\theta_{0}\left(\tau \right)\right)\overset{d}{\to }N\left(0,{\tilde{V}}_{i}\right)$ as *T* → ∞. Fix *N* and let *T* tend to infinity, then we have

$$\begin{array}{cc} \sqrt{NT}\left(\hat{\theta }\left(\tau \right)-\theta_{0}\left(\tau \right)\right) & ={\left(\frac{1}{N}\sum_{i=1}^{N}{\hat{\tilde{V}}}_{i}^{-1}\right)}^{-1}\frac{1}{\sqrt{N}}\sum_{i=1}^{N}{\hat{\tilde{V}}}_{i}^{-1}\sqrt{T}\left({\hat{\theta }}_{i}\left(\tau \right)-\theta_{0}\left(\tau \right)\right)\\ & \overset{d}{\to }{\left(\frac{1}{N}\sum_{i=1}^{N}{\tilde{V}}_{i}^{-1}\right)}^{-1}\frac{1}{\sqrt{N}}\sum_{i=1}^{N}{\tilde{V}}_{i}^{-1}N\left(0,{\tilde{V}}_{i}\right)\\ & ={\left(\frac{1}{N}\sum_{i=1}^{N}{\tilde{V}}_{i}^{-1}\right)}^{-1}\frac{1}{\sqrt{N}}\sum_{i=1}^{N}N\left(0,{\tilde{V}}_{i}^{-1}\right). \end{array}$$

Then let *N* tend to infinity, we can obtain ${\left(\frac{1}{N}\sum_{i=1}^{N}{\tilde{V}}_{i}^{-1}\right)}^{-1}\overset{p}{\to }\tilde{V}$. Moreover, by Lyapunov Central Limit Theorem, it follows that $\frac{1}{\sqrt{N}}\sum_{i=1}^{N}N\left(0,{\tilde{V}}_{i}^{-1}\right)\overset{d}{\to }N\left(0,{\tilde{V}}^{-1}\right)$. Hence, by Slutsky’s theorem, we can obtain the following result:

$$\sqrt{NT}\left(\hat{\theta }\left(\tau \right)-\theta_{0}\left(\tau \right)\right)\overset{d}{\to }N\left(0,\tilde{V}\right).$$

Following, we derive the asymptotic normality of the IV-MDQR estimator under joint asymptotics. Let Ξ = [**0**|***I***_*p*+1_]. Under assumption A8′, we can do the following computation:

$$\begin{array}{cc} & \;\;\sqrt{NT}\left(\hat{\theta }\left(\tau \right)-\theta_{0}\left(\tau \right)\right)\\ & ={\left(\frac{1}{N}\sum_{i=1}^{N}{\hat{\tilde{V}}}_{i}^{-1}\right)}^{-1}\frac{\sqrt{N}}{N}\sum_{i=1}^{N}{\hat{\tilde{V}}}_{i}^{-1}\Xi \sqrt{T}\left({\hat{\theta }}_{i}\left(\tau \right)-\theta_{0}\left(\tau \right)\right)\\ & =({\left(\frac{1}{N}\sum_{i=1}^{N}{\tilde{V}}_{i}^{-1}\right)}^{-1}+O_{p}(T^{-1/2}h_{N}^{-1/2}))(\frac{\sqrt{N}}{N}\sum_{i=1}^{N}{\tilde{V}}_{i}^{-1}\Xi \sqrt{T}\left({\hat{\theta }}_{i}\left(\tau \right)-\theta_{0}\left(\tau \right)\right)\\ & \;+O_{p}\left(N^{1/2}T^{-1/2}h_{N}^{-1/2}\right))\\ & ={\left(\frac{1}{N}\sum_{i=1}^{N}{\tilde{V}}_{i}^{-1}\right)}^{-1}\frac{\sqrt{N}}{N}\sum_{i=1}^{N}{\tilde{V}}_{i}^{-1}\Xi \sqrt{T}({\hat{\theta }}_{i}\left(\tau \right)-\theta_{0}\left(\tau \right)+O_{p}\left(N^{1/2}T^{-1/2}h_{N}^{-1/2}\right) \end{array}$$

Based on Lemmas 4 and 5 in [15], we can obtain that for each *i*,

$$\begin{array}{cc} {\hat{\theta }}_{i}\left(\tau \right)-\theta_{0}\left(\tau \right) & =-\Xi {\tilde{\Gamma }}_{i}^{-1}S_{iT}\left(\vartheta_{i0}\left(\tau \right)\right)+O_{p}\left(d_{N}\right)+O_{p}(\frac{1}{T})+\Xi {\tilde{\Gamma }}_{i}^{-1}O_{p}\left({\left({\hat{\vartheta }}_{i}\left(\tau \right)-\vartheta_{i0}\left(\tau \right)\right)}^{2}\right)\\ & =-\Xi {\tilde{\Gamma }}_{i}^{-1}S_{iT}\left(\vartheta_{i0}\left(\tau \right)\right)+O_{p}\left(d_{N}\right), \end{array}$$

where ${\tilde{\Gamma }}_{i}=\frac{\partial S_{i}\left(\vartheta_{i0}\right)}{\partial \vartheta_{i}}$, $S_{iT}\left(\vartheta \right)=\frac{1}{T}\sum_{t=1}^{T}\psi_{\tau }\left(y_{it}-\rho D_{it}-X_{it}^{⊤}\beta -\eta_{i}\right){\left[D_{it},X_{it}^{⊤},1\right]}^{⊤}$, $S_{i}\left(\vartheta \right)=E\left(S_{iT}\left(\vartheta \right)\right)$, $d_{N}=\frac{\text{log}\;\delta_{N}}{T}\vee \sqrt{\frac{\delta_{N}\left|\text{log}\;\delta_{N}\right|}{T}}$, $\delta_{N}=\sqrt{\frac{\text{log}\;N}{T}}$.

Then it follows that

$$\frac{\sqrt{N}}{N}\sum_{i=1}^{N}{\tilde{V}}_{i}^{-1}\Xi \sqrt{T}\left({\hat{\theta }}_{i}\left(\tau \right)-\theta_{0}\left(\tau \right)\right)=-\frac{\sqrt{N}}{N}{\tilde{V}}_{i}^{-1}\Xi \sqrt{T}{\tilde{\Gamma }}_{i}^{-1}S_{iT}\left(\vartheta_{i0}\left(\tau \right)\right)+O_{p}\left(\sqrt{NT}d_{N}\right).$$

As (*T*, *N*)→∞ and $\frac{N^{2}\text{log}\;N}{T}|\text{log}\frac{{\left(\text{log}\;N\right)}^{1/2}}{T^{1/2}}{|}^{2}\to 0$, the second term of the above equation is *o*_*p*_(1). Moreover, by Lyapunov central limit theorem, the first term of the above equation converges in distribution to $N(0,{\text{lim}}_{N\to }\frac{1}{N}\sum_{i=1}^{N}{\tilde{V}}_{i}^{-1})$. Hence, by Slutsky’s theorem, we can obtain the following result:

$$\sqrt{NT}\left(\hat{\theta }\left(\tau \right)-\theta_{0}\left(\tau \right)\right)\overset{d}{\to }N\left(0,\tilde{V}\right)$$

as (*T*, *N*)→∞ and $\sqrt{NT}d_{N}\to 0$.

## Supporting information

S1 Data (XLS)

S2 Data (XLS)
